# Real-world healthcare resource use and costs associated with the management of locally advanced head and neck cancer in Portugal – TRACE2 study

**DOI:** 10.3389/fonc.2026.1773554

**Published:** 2026-05-29

**Authors:** Maria Margarida Teixeira, Joana Magalhães, Joana Marinho, Katia Ladeira, Catarina Morais, Mafalda Nogueira

**Affiliations:** 1 Department of Medical Oncology, Instituto Português de Oncologia de Coimbra Francisco Gentil, Coimbra, Portugal; 2 Department of Medical Oncology, Unidade Local de Saúde do Algarve, Faro, Portugal; 3 Department of Medical Oncology, Unidade Local de Saúde Gaia e Espinho, Vila Nova de Gaia, Portugal; 4 Department of Oncology, Unidade Local de Saúde de Trás-os-Montes e Alto Douro, Vila Real, Portugal; 5 MSD Portugal, Paço de Arcos, Portugal

**Keywords:** head and neck cancer, healthcare costs, healthcare resource utilization, locally advanced disease, real-world evidence

## Abstract

**Introduction:**

Approximately 60% of patients with head and neck squamous cell carcinoma are diagnosed with locally advanced disease (LA-HNSCC), which is associated with a high risk of recurrence, distant metastasis, and poor prognosis. A multidisciplinary approach is recommended for comprehensive care, resulting in a substantial burden on the healthcare system. The TRACE2 study aimed to describe patients’ clinical characteristics, healthcare resource utilization (HCRU), and costs associated with LA-HNSCC management in real-world practice in Portugal.

**Methods:**

Non-interventional, retrospective cohort study conducted in four Portuguese hospitals between 30/11/2021-04/03/2024, including adult patients diagnosed between 01/06/2016-31/05/2018 who were eligible for curative treatment. Secondary data were collected from medical records from the time of diagnosis until recurrence, death, loss to follow-up, or the cut-off date (31/12/2020), whichever occurred first. The financial analysis was conducted from the perspective of the Portuguese public healthcare payer, considering only direct medical costs.

**Results:**

The study included 150 patients (94.7% male; 91.3% aged 50–79 years), with a high prevalence of tobacco (92.0%) and heavy alcohol (83.1%) use. Combined systemic therapy and radiotherapy (ST+RT) was the most frequent treatment (81.3%), whereas a minority underwent surgery (17.3%) or RT without ST (13.3%). During a median follow-up of 12 months, over 89% of patients required outpatient consultations and medical exams. Hospitalizations and emergency consultations were reported in 74.5% and 37.6% of patients, respectively. Overall, supportive care, including nutritional support (85.2%), psychological treatment (16.1%), and speech therapy (8.7%), were used by fewer patients. HCRU was generally higher during treatment than after its completion or discontinuation. The global median (P25; P75) annualized cost per patient was €7,706.0 (3,775.8; 20,784.8), with the primary cost drivers being ST+RT and hospitalizations, followed by outpatient consultations and imaging assessments.

**Discussion:**

The TRACE2 study provides the first comprehensive description of LA-HNSCC management in real-world practice in Portugal, underscoring the burden on both patients and the healthcare system. Given the persistently high proportion of patients diagnosed with locally advanced disease, these findings offer supporting evidence for the implementation of measures to improve patient follow-up protocols and resource allocation, ultimately enhancing patient outcomes.

## 1 Introduction

Head and neck cancers (HNC) comprise a heterogeneous group of tumors of the oral cavity, sinonasal cavity, pharynx, and larynx (1). In 2022, there were an estimated 891,453 new cases worldwide (2). Head and neck squamous cell carcinoma (HNSCC) is the most common type of HNC, arising from the mucosal surfaces of the affected anatomical sites (1). Established risk factors for HNSCC include tobacco use, alcohol consumption, and occupational exposure to carcinogens (3, 4). Notably, the rising incidence of oropharyngeal cancer in younger patients has been increasingly linked to infection with human papillomavirus (HPV) 16 (5). Combined exposure of HPV, smoking, and alcohol increases the risk for oral squamous cell carcinoma.

Approximately 60% of HNSCC cases are locally advanced (LA) at diagnosis (6, 7). LA-HNSCC is characterized by large tumors with a high risk of local recurrence and distant metastasis and is associated with a poor prognosis (1).

In LA-HNSCC, a multimodality treatment approach is generally recommended (8). The standard treatment options are either surgery combined with adjuvant chemotherapy and/or radiotherapy (RT) or primary concomitant chemoradiotherapy (9, 10). For T3/T4 cancers of the oral cavity and T4 laryngeal cancers, primary surgical treatment followed by adjuvant RT or chemoradiotherapy is usually preferred (10). Concomitant CRT is recommended for larynx preservation and for patients with unresectable tumors, or when surgery is likely to result in poor functional outcomes or prognosis (10). Transoral robotic surgery (TORS) is increasingly being used in routine clinical practice worldwide, providing a minimally invasive approach to accessing tumors in anatomically challenging areas (11). In addition, LA-HNSCC treatment should encompass not only oncologic therapies but also nutritional and swallowing assessments, dental care, and pain management (12).

The comprehensive, multidisciplinary management of HNC has substantial implications for the economic burden on healthcare systems (13). It is projected that HNC will result in a global cumulative loss of USD 535 billion in economic output between 2018 and 2030 (14).

In Portugal, the healthcare resource utilization (HCRU) and economic burden associated with LA-HNSCC are also presumed to be substantial, but real-world evidence is lacking. The TRACE-2 study aimed to provide real-world characterization of LA-HNSCC patients and assess HCRU and associated costs of disease management in Portugal.

## 2 Materials and methods

### 2.1 Study design and setting

TRACE 2 was a non-interventional, retrospective cohort study conducted in four Portuguese hospitals (Unidade Local de Saúde de Trás-os-Montes e Alto Douro – Hospital de Vila Real, Unidade Local de Saúde de Gaia e Espinho, Instituto Português de Oncologia de Coimbra, Unidade Local de Saúde do Algarve – Hospital de Faro). The study was conducted between November 30, 2021, and March 4, 2024, and collected data from the time of LA-HNSCC diagnosis (between June 1, 2016, and May 31, 2018) until recurrence, death, loss to follow-up, or the cut-off date (December 31, 2020), whichever occurred first.

### 2.2 Study population

The study included adult patients (aged ≥18 years) diagnosed with LA-HNSCC between June 1, 2016, and May 31, 2018, who were eligible for curative treatment and had primary tumors located in the oral cavity, oropharynx, hypopharynx, larynx, or other ill-defined sites within the lip, oral cavity, and pharynx. The following patients were excluded: (1) younger than 18 years; (2) with an unconfirmed LA-HNSCC diagnosis; (3) with LA-HNSCC confirmed only during autopsy; (4) with carcinoma in other anatomical sites, including nasopharynx, nasal cavity, paranasal sinuses, or salivary glands; (5) with current or previous participation in interventional clinical studies related to LA-HNSCC.

### 2.3 Study endpoints and data sources

Secondary data were collected from electronic medical records. Endpoints included patient demographics (geographic region, sex, and age), risk factors (smoking status, heavy alcohol consumption (>100 g of alcohol/day), and HPV status for patients with oropharyngeal cancer at diagnosis), clinical data (tumor location, disease stage), Eastern Cooperative Oncology Group [ECOG) performance status score, and treatment), HCRU (concomitant medication, hospitalizations, consultations, imaging assessments, laboratory tests, exams, and supportive interventions) and HCR cost information. When available, the Diagnosis Related Group (DRG) codes were collected. The cost estimation was conducted from the perspective of the public Portuguese healthcare system payer, considering only direct medical costs (15). Unit costs were calculated via micro-costing, using the Portuguese Healthcare System database (available at Portaria n.° 254/2018 | DR), which applies to all investigational centers. Costs associated with HCRU were annualized taking into account the number of months that each patient spent in the study and included medical exams, consultations, hospitalizations, emergency visits, and supportive care. HCRU costs were calculated for the whole sample and considering the patients that utilized a given healthcare resource.

### 2.4 Statistical analysis​

Quantitative variables were described using mean, standard deviation (SD), median, and first and third quartiles (P25, P75). Qualitative variables were summarized using absolute and relative frequencies. Only valid available data were analyzed, and there was no imputation of missing values. Descriptive statistics for the HCRU (use and costs) were calculated for the entire study population and subgroups. For inferential analysis, comparison of continuous variables between two groups was performed using the Mann–Whitney U test. For comparisons involving three or more groups, the Kruskal–Wallis test was applied. When a statistically significant difference was observed in analyses involving more than two groups, *post hoc* multiple comparisons were conducted using Dunn’s test for continuous variables. Categorical variables were compared between groups using the Chi-squared test or Fisher’s exact test, as appropriate based on the validity of test assumptions. When significant differences were detected in comparisons involving more than two groups, pairwise comparisons were performed using Fisher’s exact test with appropriate adjustment for multiple testing. All hypotheses were tested using two-sided tests, with a significance level set at 5%. Whenever applicable, p-values were adjusted for multiple comparisons using the Benjamini–Hochberg method, controlling the false discovery rate at 0.05. Statistical analyses were performed using the R software (version 4.2.2).

### 2.5 Ethical considerations

This study was conducted in accordance with the Declaration of Helsinki and the Good Pharmacoepidemiology Practices of the International Society for Pharmacoepidemiology (ISPE 2015). Ethical approval was obtained from the ethics committee and administration board of each participating center. Given the retrospective nature of the study, the requirement for informed consent was waived. All data were anonymized, and results are presented in aggregate form, without any patient-identifiable information.

## 3 Results

### 3.1 Sociodemographic and clinical characterization of LA-HNSCC patients

A total of 150 patients diagnosed with LA-HNSCC between 1^st^ June 2016 and 31^st^ May 2018, who were eligible for curative treatment, met the eligibility criteria and were included in this study. **Table 1** summarizes their sociodemographic and clinical characteristics. Most LA-HNSCC patients were male (94.7%) and aged between 50 and 79 years (91.3%). Over 90% of patients had a history of tobacco use, including current (48.3%) or former (45.6%) tobacco smokers, and 83.1% reported heavy alcohol consumption (> 100 g alcohol/day).

**Table 1:** Patients’ sociodemographic and clinical characteristics [n (%)].

Sociodemographic characteristics	n=150
Sex	
Male	142 (94.7)
Female	8 (5.3)
Age at study inclusion (years)	
≤39	0 (0.0)
40-49	10 (6.7)
50-59	54 (36.0)
60-69	51 (34.0)
70-79	32 (21.3)
≥80	3 (2.0)
Geographic distribution of treatment	
North	17 (11.3)
Center	102 (68.0)
South	31 (20.7)
Smoking status	
Current smoker	71 (48.3)
Former smoker	67 (45.6)
Never	9 (6.1)
*Missing*	3
Heavy alcohol consumption	
Yes	123 (83.1)
No	25 (16.9)
*Missing*	2

Clinical characteristics	*n=*150
Tumor location	
Oropharynx	57 (38.0)
Larynx	34 (22.7)
Hypopharynx	33 (22.0)
Oral cavity*	26 (17.3)
Stage at LA diagnosis	
III	19 (12.7)
IVA	113 (75.3)
IVB	18 (12.0)
T1	6 (4.1)
T2	24 (16.4)
T3	46 (31.5)
T4a	46 (31.5)
T4b	24 (16.4)
*Missing*	4
N0	19 (12.7)
N1	6 (4.0)
N2	107 (71.3)
N3	18 (12.0)
ECOG performance status	
0	108 (87.8)
1	12 (9.8)
2	3 (2.4)
*Missing*	27

HPV status in patients with OPC	*n=*57
Positive	3 (5.3)
Negative	5 (8.8)
Not tested	49 (85.9)

*Located in the inner oral mucosa, excluding the external region of the lip.

At diagnosis, 38.0% of patients presented the primary tumor in the oropharynx, 22.7% in the larynx, 22.0% in the hypopharynx, and 17.3% in the oral cavity. Most LA-HNSCC patients were diagnosed at stage IVA (75.3%), presenting with T3 (31.5%) or T4a (31.5%) tumors and N2 nodal involvement (71.3%). ECOG PS score ranged from 0 to 2, with 87.8% of patients with ECOG 0. HPV screening at diagnosis was performed in only14.1% of patients with oropharyngeal cancer.

### 3.2 Treatment patterns in LA-HNSCC patients

**Figure 1:**
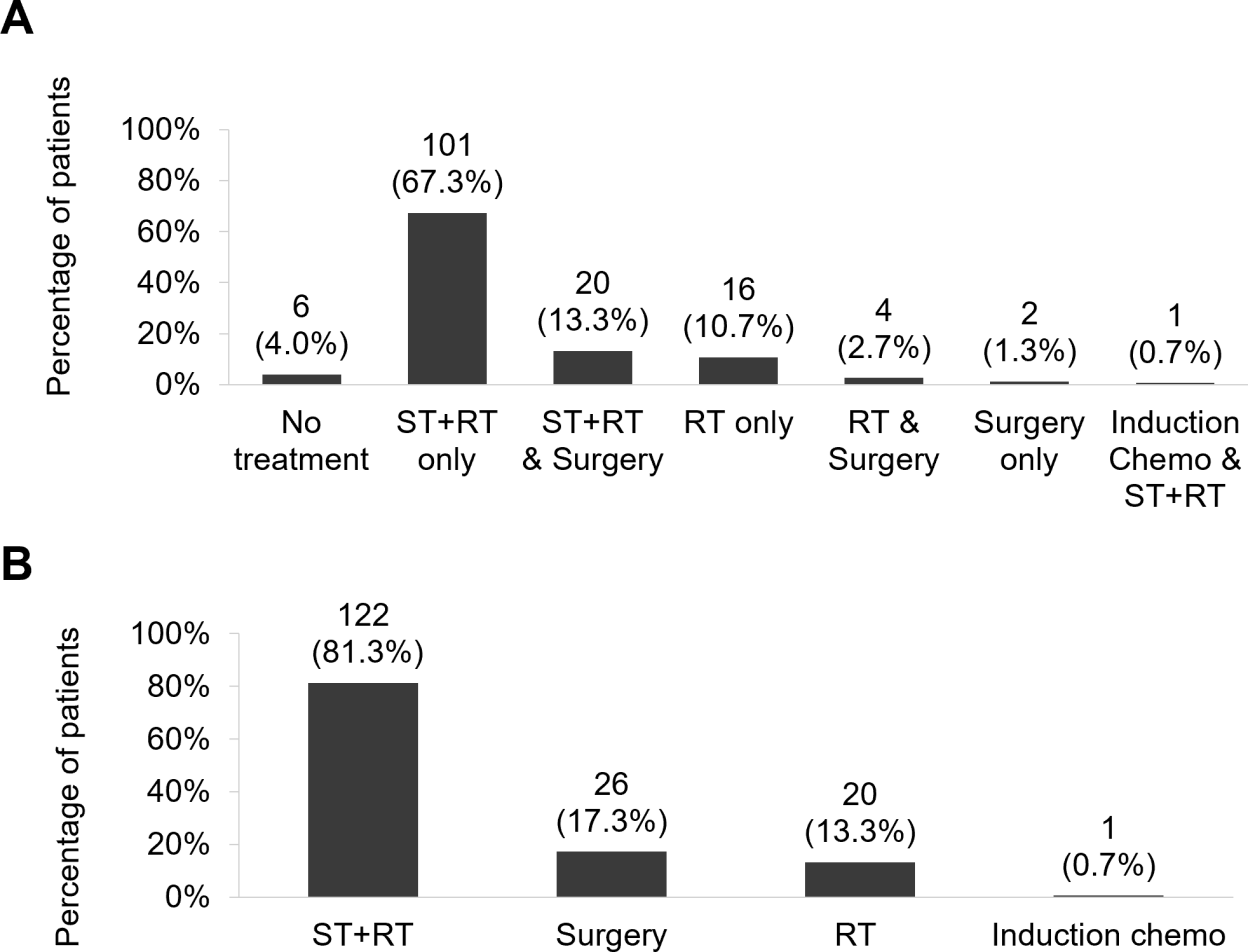
**(A)** Proportion of LA-HNSCC patients receiving different treatment combinations and **(B)** treatment modalities in LA-HNSCC patients. ST, systemic therapy; RT, radiotherapy; chemo, chemotherapy.

Treatment initiation occurred at a mean (± SD) of 4.8 (± 3.2) weeks after diagnosis, with a median of 4.0 weeks [(P25; P75): (3.0; 5.0)]. The duration of treatment averaged 7.6 (± 3.3) weeks, with a median of 7.0 weeks [(P25; P75): (7.0; 7.0)], ranging from 1 to 29 weeks. The distribution of treatment combinations is presented in **Figure 1A**. Systemic therapy (ST) combined with radiotherapy (RT) was administered to 67.3% of patients (*n=*101), 13.3% (*n=*20) received ST+RT in combination with surgery, 10.7% (*n=*16) were treated with RT alone, 2.7% (*n=*4) were treated with RT and surgery, 1.3% (*n=*2) underwent surgery alone, and 0.7% (*n*=1) received induction chemotherapy followed by ST+RT. Notably, 4.0% of patients (*n*=6) did not receive any treatment, due to rapid disease progression or death. Overall, 81.3% (*n=*122) were treated with ST+RT, 17.3% (*n=*26) underwent surgery, and 13.3% (*n=*20) received RT without ST (**Figure 1B**). In patients receiving ST+RT, cisplatin was predominantly used (95.9%), with a median cumulative dose of 170.0 [160.0; 180.0] mg/m^2^ administered over a median of 2.0 [2.0; 3.0] cycles, combined with RT that was delivered to a total median dose of 70.0 Gy [70.0; 70.0] in a median of 33.0 [33.0; 33.0] sessions (**Supplementary Table 1**). Patients who received RT without ST were administered the same median RT dose and number of sessions (**Supplementary Table 1**). Induction chemotherapy consisted of one cycle of 75.0 mg/m^2^ cisplatin combined with 750 mg/m^2^ 5-FU (**Supplementary Table 1**). All patients who underwent surgery received a single surgical procedure (**Supplementary Table 1**). The most frequent surgical procedures were excision (84.6%) and lymph node dissection (80.8%) (**Supplementary Table 2**).

### 3.3 Use of healthcare resources by LA-HNSCC patients

Patients were prescribed concomitant medication, underwent complementary medical exams, attended consultations, and received specialized medical care both during treatment and after treatment completion or discontinuation.

The majority of patients attended outpatient consultations (98.7%), both during treatment (98.7%) and after treatment completion or discontinuation (85.9%) (**Table 2**). Overall, patients attending consultations were seen by medical oncologists (96.6%), other specialists (100%), and multidisciplinary teams (88.4%). While consultations with medical oncologists and multidisciplinary teams were frequent during treatment (96.6% and 86.4%, respectively), their frequency declined after treatment (50.8% and 49.2%, respectively). **Supplementary Table 3** provides information on the number of appointments per patient.

**Table 2:** Healthcare resource utilization in the care of LA-HNSCC patients, overall and during or after treatment completion or discontinuation [n (%)].

Type of HCR	Global use	During treatment	After treatment
Outpatient consultations*, missing n=1*	147 (98.7)	147 (98.7)	128 (85.9)
Medical oncology	142 (96.6)	142 (96.6)	65 (50.8)
Other medical specialties	147 (100.0)	143 (97.3)	124 (96.9)
Multidisciplinary team	130 (88.4)	127 (86.4)	63 (49.2)
Exams, missing n=1			
Imaging	149 (100.0)	143 (96.0)	110 (73.8)
Laboratory	149 (100.0)	149 (100.0)	86 (57.7)
Biopsies, ECG, and others	134 (89.9)	128 (85.9)	47 (31.5)
Hospitalizations, *missing n=1*	111 (74.5)	77 (51.7)	65 (43.6)
Emergency consultations*, missing n=1*	56 (37.6)	39 (26.2)	37 (24.8)
Supportive care*, missing n=1*			
Concomitant medication	141 (94.6)	138 (92.6)	95 (63.8)
Nutritional support	127 (85.2)	127 (85.2)	70 (47.0)
Psychological treatment	24 (16.1)	15 (10.1)	14 (9.4)
Speech therapy	13 (8.7)	7 (4.7)	10 (6.7)
Physiatry, pain consultation, rehabilitation, tracheostomy, other	N.A.	N.A.	18 (12.1)

Complementary medical exams were also widely used, with all patients undergoing imaging and laboratory tests, and 89.9% performing biopsies, ECG, or other exams (**Table 2**). The use of these resources occurred primarily during treatment and declined considerably thereafter. The number of exams performed per patient is detailed in **Supplementary Table 3**.

Hospitalization was required for 74.5% of patients, with similar rates observed during (51.7%) and after (43.6%) treatment (**Table 2**). Emergency consultations were less frequent, occurring in 37.6% of patients overall and in approximately 25-26% of cases during both periods (**Table 2**). The number of hospitalizations and emergency visits per patient is listed in **Supplementary Table 3**.

Although nearly all patients (94.6%) were prescribed concomitant medication for disease management during the study period (**Table 2**), prescription patterns were different during and after treatment. During systemic treatment, 92.6% of patients received concomitant medications. Among these (*n=*138), the most frequently prescribed included anti-emetics (*n=*102, 73.9%), grade 1 or 2 (non-opioid) analgesics (*n=*99, 71.7%), IV hydration (*n=*98, 71.0%), nutritional supplement - tube feeds and nutritional drinks (*n=*92, 66.7%), and grade 3 (opioid) analgesics (*n=*79, 57.2%). After treatment completion or discontinuation, the proportion of patients receiving medication decreased to 63.8%. Among them (*n=*95), 60.0% (*n=*57) were prescribed grade 1 or 2 (non-opioid) analgesics, 51.6% (*n=*49) grade 3 (opioid) analgesics, and 44.2% (*n=*42) nutritional supplements.

Global utilization of nutritional support was high (85.2%), with 85.2% of patients receiving it during treatment; however, its use declined to 47.0% after treatment (**Table 2**). In contrast, only a small proportion of patients received psychological treatment (16.1%) and speech therapy (8.7%) (**Table 2**). Additional supportive care after treatment, including physiatry, pain consultation, rehabilitation, and tracheostomy, was documented in 12.1% of patients (**Table 2**). **Supplementary Table 3** presents the number of consultations per patient for each supportive service.

### 3.4 Palliative care in patients with locoregional recurrence or metastasis

This study included a median follow-up duration of 12.0 [5.0; 30.5] months. Reasons for the end of data collection comprised reaching the cut-off date (*n=*42, 28.2%), death (*n=*36, 24.2%), locoregional recurrence (*n=*31, 20.8%), metastasis (*n=*31, 20.8%), and loss to follow-up (*n=*9, 6.0%).

The use of palliative care was assessed in cases of recurrent or metastatic disease and not at locally advanced stage. Among the 62 LA-HNSCC patients who experienced locoregional recurrence or distant metastasis during the study period (**Table 3**), 43.5% received palliative care. Of these, 77.8% received palliative care in a hospital setting, 14.8% in specialized facilities, and 7.4% at home. The choice of care setting was influenced by various factors, with the most common being insufficient resources for the patients’ preferred setting (37.0%) and personal preference (29.6%). In 68.6% of cases among patients who did not receive palliative care, the primary reason was ineligibility for admission to the palliative care unit due to the initiation of treatment for recurrent or metastatic disease, with fewer cases attributed to other factors or death. Additionally, 25.7% of patients lacked access to palliative care services.

**Table 3:** Palliative care use by LA-HNSCC patients with locoregional recurrence or distant metastasis [n (%)].

Palliative care	n=62
Yes	27 (43.5)
No	35 (56.5)
Palliative Care Setting	
Hospital	21 (77.8)
Specialized Palliative Care Facility	4 (14.8)
Home	2 (7.4)
Palliative Care Setting determinants	
Insufficient resources for palliative care setting of choice	10 (37.0)
Patient preference	8 (29.6)
Direct referral, rapid deterioration, or other	7 (25.9)
No access to palliative care setting of choice	2 (7.4)
Reason for no palliative care	
Ineligibility for admission in palliative care unit, death, or other	24 (68.6)
Lack of access	9 (25.7)
No need	2 (5.7)
Patient refusal	0 (0.0)
Insufficient resources	0 (0.0)

### 3.5 Cost of healthcare resources used by LA-HNSCC patients​

Costs associated with HCRU were annualized taking into account the number of months that each patient spent in the study and included medical exams, consultations, hospitalizations, emergency visits, and supportive care. HCRU costs were calculated for the whole sample and considering the patients that utilized a given healthcare resource. Although data on concomitant medication were collected, the database lacked information to determine its costs. The overall mean/median annualized cost per patient for healthcare resources associated with LA-HNSCC patient care during follow-up was €20,774.4 (± 3,527.4)/€7,706.0 [3,775.8; 20,784.8]. The mean/median annualized costs per patient were higher during treatment [€13,213.5 (± 1,587.1)/€ 5,721.8 (1,984.0; 15,303.8)] than after its completion or discontinuation [€7,575.4 (± 2,364.6)/€1,640.6 (450.2; 4,207.3)]. The mean and median overall annualized costs of each healthcare resource per patient, both for the entire study sample and for the subgroup using each specific resource, are summarized in **Table 4**. ST+RT and hospitalization were the costliest healthcare resources, with median per-patient annualized costs of €2,503.1 [779.0; 6,969.2] and €2,305.3 [0.0; 9,880.0], respectively. Although lower in value, outpatient consultations also contributed substantially to the overall per-patient annualized cost [€806.0 (420.0; 2,099.1)], as did imaging assessments [€532.6 (200.0; 1,287.8)]. The costs associated with supportive care were amongst the lowest of all healthcare resources. **Supplementary Table 4** provides a detailed breakdown of costs during and after treatment.

**Table 4:** Overall annualized cost per patient (€) of each HCR used in the management of LA-HNSCC, considering the study population or only the subgroup using the HCR.

Overall annualized cost *per* patient	Study population	Subgroup using HCR
Treatment		
ST + RT	*n=*150	*n=*122
Median [P25; P75]	2,503.1 [779.0; 6,969.2]	3,533.7 [1,230.9; 8,413.6]
Mean (SD)	4,507.1 (6,602.5)	5,878.9 (6,989.5)
*Missing*	30	30
Surgery	*n=*150	*n=*26
Median [P25; P75]	0 [0; 0]	782.4 [260.8; 3,759.1]
Mean (SD)	1,093.8 (6,329.2)	6,268.1 (14,262.4)
*Missing*	1	0
RT	*n=*150	*n=*20
Median [P25; P75]	0 [0; 0]	3,794.4 [964.1; 7,557.5]
Mean (SD)	546.2 (1,868.2)	4,096.6 (3,472.8)
Induction chemotherapy	*n=*150	*n=*1
Median [P25; P75]	0 [0; 0]	34.5 [34.5; 34.5]
Mean (SD)	0.2 (2.8)	34.5 (NA)
Outpatient consultations	*n=*150	*n=*147
Median [P25; P75]	806.0 [420.0; 2,099.1]	806.0 [436.8; 2,139.0]
Mean (SD)	1,512.7 (1,697.4)	1,533.3 (1,699.6)
*Missing*	1	0
Exams		
Imaging	*n=*150	*n=*149
Median [P25; P75]	532.6 [200.0; 1,287.8]	532.6 [200.0; 1,287.8]
Mean (SD)	998.0 (1,522.1)	998.0 (1,522.1)
*Missing*	1	0
Laboratory	*n=*150	*n=*149
Median [P25; P75]	84.0 [40.0; 218.1]	84.0 [40.0; 218.1]
Mean (SD)	177.4 (259.5)	177.4 (259.5)
*Missing*	1	0
Biopsies, ECG, and others	*n=*150	*n=*134
Median [P25; P75]	70.8 [26.6; 141.7]	85.0 [35.4; 141.7]
Mean (SD)	173.0 (404.3)	192.3 (422.0)
*Missing*	1	0
Hospitalizations	*n=*150	*n=*111
Median [P25; P75]	2,305.3 [0.0; 9,880.0]	3,458.0 [1,753.7; 13,020.4]
Mean (SD)	9,926.2 (2,074.6)	13,324.3 (2,713.2)
*Missing*	1	0
Emergency consultations	*n=*150	*n=*56
Median [P25; P75]	0 [0; 66.2]	102.0 [55.6; 354.1]
Mean (SD)	110.0 (299.1)	292.6 (431.7)
*Missing*	1	0
Supportive care		
Nutritional support	*n=*150	*n=*125
Median [P25; P75]	106.7 [27.4; 240.0]	144.0 [57.6; 296.7]
Mean (SD)	190.0 (282.4)	226.4 (294.7)
*Missing*	1	0
Psychological treatment	*n=*150	*n=*24
Median [P25; P75]	0 [0; 0]	49.0 [26.2; 102.9]
Mean (SD)	15.7 (73.9)	97.4 (163.8)
*Missing*	1	0
Speech therapy	*n=*150	*n=*12
Median [P25; P75]	0 [0; 0]	32.9 [13.8; 57.0]
Mean (SD)	3.6 (16.6)	44.4 (41.8)
*Missing*	1	0
Physiatry, pain consultation, rehabilitation, tracheostomy, other	*n=*150	*n=*18
Median [P25; P75]	0 [0; 0]	89.0 [33.8; 223.2]
Mean (SD)	2,389.2 (19,663.1)	20,940.8 (56,457.3)
*Missing*	1	1

### 3.6 Healthcare utilization and associated cost according to tumor location and surgery status

Next, the association between HCRU with tumor location was evaluated (**Table 5**). Regarding treatment patterns, statistically significant differences were observed in the proportion of patients undergoing surgery. Specifically, patients with tumors located in the oropharynx underwent surgical treatment significantly less frequently (1.8%) than those with tumors in the oral cavity (30.8%; *p=*0.0008), hypopharynx (21.2%; *p=*0.0066), and larynx (29.4%; *p=*0.0008), while no significant differences were identified for other treatment modalities. The utilization of imaging assessments, laboratory tests, and other examinations did not vary significantly by tumor location. Similarly, tumor location was not associated with differences in hospitalization rates or outpatient consultations. However, patients with laryngeal tumors required emergency room consultations significantly more often than those with tumors in the oral cavity (55.9% *vs.* 15.4%; *p=*0.0093). To assess whether observed differences in utilization translated into cost variation, costs were evaluated for resources with differential use (**Table 6**). No significant differences were observed in these costs (*i.e.*, surgery and emergency consultations), consistent with the lack of differences in median total annualized costs across tumor location groups.

**Table 5:** Healthcare resource utilization in the care of LA-HNSCC patients according to tumor location [n (%)].

Type of HCR	Oral cavity (n=26)	Hypopharynx (n=33)	Oropharynx (n=57)	Larynx (n=34)	Adjusted p value
ST+RT	19 (73.1)	25 (75.8)	52 (91.2)	26 (76.5)	0.1881 (Fisher’s exact)
RT (no ST)	5 (19.2)	6 (18.2)	3 (5.3)	6 (17.6)	0.2390 (Fisher’s exact)
Surgery	8 (30.8)	7 (21.2)	1 (1.8)^*^	10 (29.4)	**0.0192** (Fisher’s exact) Multiple Comparison: Fisher’s exact Hypopharynx vs oral cavity p=0.6916 Oral cavity vs oropharynx p=**0.0008** Larynx vs oral cavity p=1.0000 Hypopharynx vs oropharynx p=**0.0066** Hypopharynx vs larynx p=0.6916 Larynx vs oropharynx p=**0.0008**
Outpatient Consultations	26 (100.0)	32 (97.0)	56 (100.0)^*^	33 (97.1)	0.4889 (Fisher’s exact)
Imaging assessments	26 (100.0)	33 (100.0)	56 (100.0)^*^	34 (100.0)	-–-
Laboratory	26 (100.0)	33 (100.0)	56 (100.0)^*^	34 (100.0)	-–-
Biopsies, ECG, and others	22 (84.6)	33 (100.0)	48 (85.7)^*^	31 (91.2)	0.1881 (Fisher’s exact)
Hospitalization	17 (65.4)	27 (81.8)	39 (69.6)^*^	28 (82.4)	0.4284 (Fisher’s exact)
Emergency consultations	4 (15.4)	9 (27.3)	24 (42.9)^*^	19 (55.9)	**0.0433** (Fisher’s exact) Multiple Comparison: Fisher’s exact Hypopharynx vs oral cavity p=0.3513 Oral cavity vs oropharynx p=0.0515 Larynx vs oral cavity **p=0.0093** Hypopharynx vs oropharynx p=0.2637 Hypopharynx vs larynx p=0.0515 Larynx vs oropharynx p=0.3349
Nutritional support	22 (84.6)	32 (97.0)	49 (87.5)^*^	24 (70.6)	0.1061 (Fisher’s exact)
Psychological treatment	2 (7.7)	4 (12.1)	12 (21.4)^*^	6 (17.6)	0.4889 (Fisher’s exact)
Speech therapy	2 (7.7)	3 (9.1)	1 (1.8)^*^	7 (20.6)	0.1061 (Fisher’s exact)

*n=1 missing Bold p-values indicate statistical significance.

**Table 6:** Total annualized cost per patient, cost of surgery, and emergency consultations costs by tumor location (€).

Annualized cost per patient		Oral cavity (n=26)	Hypopharynx (n=33)	Oropharynx (n=57)	Larynx (n=34)	Adjusted P value
Total annualized cost						
Median [P25; P75]		11,044.0 [4,062.0; 21,363.0]	15,309.0 [6,701.0; 36,347.0]	7,588.9 [3,293.2; 17,635.0]	11,740.0 [5,081.0; 48,209.0]	0.2390 (Kruskal-Wallis)
Mean (SD)		28,261.0 (66,169.4)	29,871.0 (52,648.6)	13,257.0 (13,681.6)	32,547.0 (52,972.0)	
Annualized cost of surgery						
Study population	Median [P25; P75]	0.0 [0.0; 521.9]	0.0 [0.0; 0.0]	0.0 [0.0; 0.0]	0.0 [0.0; 87.5]	-–-
	Mean (SD)	413.4 (1,163.4)	2,463.6 (10,644.4)	5.5 (41.4)	2,076.9 (7,969.5)
	*Missing*	0	0	1	0
Subgroup using the HCR	Median [P25; P75]	782.4 [692.8; 929.0]	3,917.9 [1,109.5; 7,116.5]	309.7 [309.7; 309.7]	315.8 [118.0; 2,849.1]	0.4681 (Kruskal-Wallis)
	Mean (SD)	1,343.6 (1,845.4)	11,614.1 (21,915.57)	309.7 (NA)	7,061.4 (13,920.1)
Annualized cost of emergency consultations						
Study population	Median [P25; P75]	0.0 [0.0; 0.0]	0.0 [0.0; 11.3]	0.0 [0.0; 72.9]	47.4 [0.0; 260.1]	-–-
	Mean (SD)	65.5 (228.6)	65.4 (220.8)	77.9 (148.1)	240.0 (505.0)
	*Missing*	0	0	1	0
Subgroup using the HCR	Median [P25; P75]	331.5 [43.2; 714.0]	102.0 [39.5; 174.9]	96.9 [55.6; 306.0]	122.4 [71.3; 612.0]	0.5931 (Kruskal-Wallis)
	Mean (SD)	425.7 (480.7)	239.9 (384.6)	181.7 (181.0)	429.5 (617.8)

The association between HCRU and surgery status was also evaluated, with no differences observed between patients who underwent surgery and those who received only non-surgical treatment (**Supplementary Table 5**). Consistent with these findings, no significant differences were observed in median total annualized costs (€6,701.2 [4,171.0; 17,757.0] vs €7,716.2 [3,458.9; 20,784.8] in surgical and non-surgical patients, respectively; *p=*1.000).

## 4 Discussion

The TRACE2 study provides a detailed real-world characterization of LA-HNSCC patients, as well as HCRU utilization and associated costs across four Portuguese hospitals, underscoring the burden of this disease on both patients and the healthcare system. The predominance of male patients in the study population aligns with previous international and national data on HNC (16–18). Over 80% of patients were current or former smokers and heavy alcohol consumers, both of which are well-established risk factors for HNSCC development (19). HPV exposure is a recognized risk factor for OPC (20). However, at the time this study was conducted, HPV screening in OPC patients was not routinely performed. The data presented reflect prevailing clinical practice at the time and are not intended to assess a potential association between HPV infection and OPC incidence in these patients.

The optimal treatment strategy for LA-HNSCC patients should be determined by a multidisciplinary team and tailored to factors such as disease extent, tumor location, the patient’s overall health, comorbidities, and potential impact on quality of life. Systemic therapy combined with RT was the most frequent treatment, preferentially with cisplatin, while a smaller fraction of patients underwent RT or surgery. Only one patient received induction chemotherapy with cisplatin and 5-FU, which is consistent with the lack of consensus regarding benefits over the standard of care (21). These treatment patterns are aligned with the NCCN and EHNS–ESMO–ESTRO guidelines available at the time, which recommended chemotherapy and concurrent radiotherapy for patients with unresectable, locally advanced disease (9, 22). Concomitant chemotherapy, with cisplatin, improves locoregional control and overall survival, regardless of tumor location in the oral cavity, pharynx, or larynx (23–25). Additionally, aligning with international guidelines, patients with advanced oropharyngeal tumors were preferentially managed with non-surgical treatment modalities (10). A multidisciplinary team of dedicated professionals should be involved in the care of LA-HNSCC patients to manage and prevent sequelae after treatment (12). In line with the objectives of this study, HCRU by patients with LA-HNSCC was assessed. Concomitant medication, imaging assessments, laboratory tests, and other exams, including biopsies and ECG, were utilized by most patients, particularly during treatment. Nevertheless, the utilization of these resources generally declined after treatment completion or discontinuation.

Globally, over 70% of patients required at least one hospitalization, and more than 35% of patients had at least one emergency visit during the study period, reflecting the high burden of this disease. While patients with laryngeal tumors exhibited higher rates of emergency consultations, statistically significant differences were observed only in comparison with patients with oral cavity tumors, who had the lowest rates. Although studies formally assessing differences in emergency consultations according to tumor location are limited, the symptom profile typically associated with laryngeal HNSCC (e.g., airway obstruction, tracheostomy-related complications, and the need for urgent airway interventions) may partially explain the higher emergency consultation rates observed in this group. Notably, while nearly all patients attended outpatient consultations with medical oncologists and a multidisciplinary team during treatment, only about half continued to consult with these specialists after treatment completion or discontinuation. It was not possible to assess consultations with general practitioners, an important limitation given the importance of primary care in coordinating the care of the disease and other comorbidities in LA-HNSCC patients. In government-funded systems such as Portugal’s, primary care is often the first and most accessible point of care, particularly when access to oncology follow-up is limited or delayed.

Nutritional support is crucial for patients with LA-HNSCC, as it helps optimize treatment outcomes, minimize treatment-related complications, and enhances overall quality of life (26, 27). Patients should also receive speech rehabilitation following treatment completion, as treatment often affects speech and swallowing function (28). Additionally, alterations in speech and swallowing abilities, combined with other disease-related factors, can profoundly impact patients’ psychological well-being. Although patients with HNSCC present elevated levels of psychological distress, only a minority of them express a desire for psychological care (29). In this study, most patients received nutritional support during active treatment; however, fewer than half continued this intervention in the post-treatment period. Moreover, only a small fraction of patients received speech therapy, psychological support, pain management, or rehabilitation consultations. Non-opioid and opioid analgesics were utilized by more than half of the patients on medication during and following the treatment period. This observation warrants further consideration, as pain represents a prevalent symptom arising from both the tumor itself and oncological treatment, often requiring opioid analgesics for adequate management (30). Overall, the results suggest a potential underutilization of supportive care services, although underreporting cannot be categorically excluded. These findings may help define strategies for a comprehensive assessment of unmet needs in supportive care delivery within the Portuguese clinical context, to better assist patients in managing the substantial physical and psychological burden associated with LA-HNSCC treatment.

Palliative care, which encompasses symptom management and psychosocial support throughout the disease continuum, plays a vital role in supporting LA-HNSCC patients in managing disease and treatment-related symptoms and addressing psychological distress (31). Despite evidence supporting early integration, palliative care delivery is often inappropriately delayed (32). In this study, over half of the patients who experienced recurrence or metastasis did not receive palliative care, primarily due to initiation of treatment for recurrent or metastatic disease, highlighting the complexity of care in a rapidly progressing condition.

This study provides evidence on the economic burden associated with LA-HNSCC management in Portugal. It is important to note that this study was conducted in public, government-funded hospitals; therefore, the reported costs reflect expenditures by the healthcare system that are not incurred by patients. The total mean/median annualized cost incurred during patient follow-up was estimated at €20,774.4/€7,706.0 per patient, with the primary cost drivers being ST+RT and hospitalizations, followed by outpatient consultations and imaging assessments. Conversely, the estimated costs per patient for supportive care were among the lowest across all healthcare resources. Considering that these were also among the least utilized resources, increasing their utilization is unlikely to substantially increase the economic burden on the healthcare system.

Although comparisons of healthcare costs between countries are relevant, they remain challenging due to differences in study design, the variables included in cost analyses, and variations in the unit costs of consumables and procedures. Nonetheless, the estimated mean ± SD total annualized cost per patient in this study was €20,774.4 ± €3,527.4, whereas the total cost over three years in a study conducted in France, which included palliative care and medical transportation to and from the hospital, unlike the present study, was €46,791 ± €34,841 (33).

This study has several limitations that warrant consideration. An important limitation is that 102 of the 150 patients were recruited from a single hospital, increasing the risk of selection bias and potentially limiting the representativeness of all LA-HNSCC cases in Portugal. Secondly, the observational design of this study and reliance on medical records for data collection resulted in incomplete, missing, or poorly recorded information. Moreover, as per the study design, key variables such as comorbidities, socioeconomic factors, and quality of life were not evaluated. This reflects the fact that they were outside the scope of the study objectives, although it cannot be excluded that the lack of data on these variables may limit the interpretation of the study results. These limitations may prevent our findings from fully representing the clinical complexity of LA-HNSCC in Portugal. Third, several cost-related limitations affected the accuracy of the cost estimates. Specifically, costs associated with concomitant medications, palliative care services, medical transportation to and from healthcare facilities, and indirect costs were not included in the analysis. Additionally, when the exact cost of a certain healthcare resource could not be obtained due to either the availability of multiple cost options for the same resource or the inability to identify the specific diagnosis-related group (DRG), a conservative approach was followed by using the lowest values. Lastly, although costs were annualized to account for differences in follow-up duration among patients, a median follow-up of 12 months may be insufficient to capture the disease burden. Thus, these results represent conservative estimates over a relatively short period of time and underestimate the true economic burden associated with LA-HNSCC management in the Portuguese healthcare system.

Nevertheless, the TRACE-2 study provides the first comprehensive characterization of LA-HNSCC patients, treatment patterns, HCRU, and associated costs in Portugal, demonstrating the substantial clinical and economic burden of the disease. Importantly, because the economic burden is underestimated in our analysis, the true impact of LA-HNSCC is even greater. Given the persistently high proportion of patients diagnosed with locally advanced disease (34–36), these findings offer valuable evidence to inform the implementation of measures aimed at improving patient follow-up protocols and optimizing healthcare resource allocation within the Portuguese clinical context. Such improvements could ultimately enhance patient outcomes and quality of life.

## Data Availability

The original contributions presented in the study are included in the article/**Supplementary Material**. Further inquiries can be directed to the corresponding author.
