# Supplementary material for: Real-world healthcare resource use and costs associated with the management of locally advanced head and neck cancer in Portugal – TRACE2 study
Source: Front Oncol. 2026 May 29;16:1773554. doi: 10.3389/fonc.2026.1773554 (PMC13260713; doi:10.3389/fonc.2026.1773554)
Supplement: Supplementary file 1 [file Table1.docx]

Supplementary Material

# Supplementary Figures and Tables

Table S1. Number of sessions/procedures and dose of chemotherapy, radiotherapy, and surgery used in LA-HNSCC patients.

| **Treatment use *per* patient** |  |
| --- | --- |
| **Systemic therapy + RT** | ***n=*122** |
| Number of RT sessions |  |
| Median [P25; P75] | 33.0 [33.0; 33.0] |
| Mean (SD) | 32.2 (3.9) |
| Dose (Gy) |  |
| Median [P25; P75] | 70.0 [70.0; 70.0] |
| Mean (SD) | 67.6 (8.0) |
| Cisplatin | *n=*117 |
| Dose (mg/m^2^) |  |
| Median [P25; P75] | 170.0 [160.0; 180.0] |
| Mean (SD) | 168.9 (15.5) |
| *Missing* | 26 |
| Number of cycles |  |
| Median [P25; P75] | 2.0 [2.0; 3.0] |
| Mean (SD) | 2.5 (1.3) |
| Cetuximab | *n=*5 |
| Dose (mg/m^2^) | 3243 |
| *Missing* | 4 |
| Number of cycles |  |
| Median [P25; P75] | 8.0 [7.0; 8.0] |
| Mean (SD) | 6.6 (2.6) |
| **Surgery** | ***n=*26** |
| Number of surgeries |  |
| Median [P25; P75] | 1.0 [1.0; 1.0] |
| Mean (SD) | 1.0 (0) |
| **Radiotherapy** | ***n=*20** |
| Number of sessions |  |
| Median [P25; P75] | 33.0 [33.0; 33.0] |
| Mean (SD) | 26.9 (13.2) |
| Dose (Gy) |  |
| Median [P25; P75] | 70.0 [65.2; 70.0] |
| Mean (SD) | 58.7 (24.4) |
| **Induction chemotherapy** | ***n=*1** |
| Cisplatin |  |
| Dose (mg/m^2^) | 75.0 |
| Number of cycles | 1.0 |
| 5-FU |  |
| Dose (mg/m^2^) | 750.0 |
| Number of cycles | 1.0 |

Table S2. Surgical procedures performed in LA-HNSCC patients (n (%)).

| **Surgical procedure** | ***n=*26** |
| --- | --- |
| Excision | 22 (84.6%) |
| Lymph node dissection | 21 (80.8%) |
| Tracheostomy | 6 (23.1%) |
| Reconstructive surgery | 3 (11.5%) |
| Laser | 1 (3.8%) |

Table S3. Per patient utilization of each healthcare resource in LA-HNSCC, overall and during or after treatment completion or discontinuation.

| **HCR *per* patient** | **Global use** | **During treatment** | **After treatment** |
| --- | --- | --- | --- |
| **Outpatient consultations** |  |  |  |
| Number of consultations with medical oncology |  |  |  |
| Median [P25; P75] | 6.0 [4.0; 10.0] | 5.0 [3.0; 6.0] | 6.0 [3.0; 10.0] |
| Mean (SD) | 8.6 (8.1) | 5.4 (5.0) | 6.9 (5.3) |
| Number of consultations with other medical specialties |  |  |  |
| Median [P25; P75] | 20.0 [11.0; 31.5] | 10.0 [6.5; 16.0] | 9.0 [4.0; 20.2] |
| Mean (SD) | 23.3 (16.2) | 12.4 (9.0) | 13.4 (12.2) |
| Number of consultations with multidisciplinary team |  |  |  |
| Median [P25; P75] | 2.0 [2.0; 3.8] | 2.0 [2.0; 2.0] | 2.0 [1.0; 3.0] |
| Mean (SD) | 3.1 (2.9) | 2.0 (2.1) | 2.3 (2.4) |
| **Hospitalizations** |  |  |  |
| Number of hospitalizations |  |  |  |
| Median [P25; P75] | 1.0 [1.0; 2.0] | 1.0 [1.0; 1.0] | 1.0 [1.0; 2.0] |
| Mean (SD) | 1.7 (1.1) | 1.2 (0.5) | 1.5 (1.1) |
| Number of days hospitalized |  |  |  |
| Median [P25; P75] | 21.0 [8.0; 41.0] | 20.0 [7.0; 44.0] | 10.0 [7.0; 19.0] |
| Mean (SD) | 27.2 (23.2) | 26.2 (22.7) | 15.4 (15.8) |
| **Emergency consultations** |  |  |  |
| Number of emergency consultations |  |  |  |
| Median [P25; P75] | 3.0 [1.0; 5.0] | 1.0 [1.0; 3.5] | 2.0 [1.0; 4.0] |
| Mean (SD) | 3.4 (2.8) | 2.4 (2.3) | 2.6 (2.0) |
| **Supportive care** |  |  |  |
| Number of sessions with nutrition specialist |  |  |  |
| Median [P25; P75] | 9.0 [6.0; 12.0] | 6.0 [4.0; 9.0] | 3.0 [1.0; 5.0] |
| Mean (SD) | 8.9 (4.9) | 6.9 (3.9) | 3.6 (2.7) |
| *Missing* | 2 | 0 | 0 |
| Number of psychological treatment consultations |  |  |  |
| Median [P25; P75] | 2.0 [1.0; 3.0] | 1.0 [1.0; 2.0] | 1.0 [1.0; 4.2] |
| Mean (SD) | 2.7 (2.7) | 1.7 (1.0) | 2.8 (2.8) |
| Number of speech therapy consultations |  |  |  |
| Median [P25; P75] | 4.0 [1.8; 12.5] | 1.0 [1.0; 4.0] | 3.0 [2.0; 12.0] |
| Mean (SD) | 10.1 (14.5) | 2.4 (1.8) | 11.6 (16.0) |
| *Missing* | 1 | 0 | 1 |

**Table S4**. Annualized cost per patient (€) of each HCR used during and after treatment completion or discontinuation, considering the study population or only the subgroup using the HCR.

|  | **Study population** | | **Subgroup using HCR** | |
| --- | --- | --- | --- | --- |
| **Annualized cost *per* patient, €** | **During treatment** | **After treatment** | **During treatment** | **After treatment** |
| **Outpatient consultations** | ***n=*150** | ***n=*150** | ***n=*147** | ***n=*128** |
| Median [P25; P75] | 507.3 [192.0; 1,209.0] | 315.6 [82.7;797.1] | 520.8 [196.7;1,236.9] | 394.3 [231.7;967.2] |
| Mean (SD) | 929.1 (1,138.0) | 583.6 (747.4) | 941.8 (1,140.5) | 679.4 (765.1) |
| *Missing* | 1 | 1 | 0 | 0 |
| **Exams** |  |  |  |  |
| Imaging | ***n=*150** | ***n=*150** | ***n=*143** | ***n=*110** |
| Median [P25; P75] | 219.1 [73.7;501.1] | 166.2 [0;470.5] | 227.8 [82.9;508.1] | 273.1 [108.8;1,011.4] |
| Mean (SD) | 406.4 (594.9) | 591.6 (1,277.0) | 423.4 (601.4) | 801.3 (1429.9) |
| *Missing* | 1 | 1 | 0 | 0 |
| Laboratory | ***n=*150** | ***n=*150** | ***n=*149** | ***n=*87** |
| Median [P25; P75] | 53.7 [30.0;134.0] | 14.0 [0;52.4] | 53.7 [30.0;134.0] | 42.9 [22.9;99.7] |
| Mean (SD) | 120.9 (190.2) | 56.5 (132.2) | 120.9 (190.2) | 96.8 (161.7) |
| *Missing* | 1 | 1 | 0 | 0 |
| Biopsies, ECG, and others | ***n=*150** | ***n=*150** | ***n=*128** | ***n=*54** |
| Median [P25; P75] | 42.5 [10.1;85.0] | 0 [0;35.4] | 58.7 [23.0;85.0] | 60.7 [33.4;122.4] |
| Mean (SD) | 129.6 (381.1) | 43.4 (105.1) | 150.8 (407.5) | 119.8 (146.7) |
| *Missing* | 1 | 1 | 0 | 0 |
| **Hospitalizations** | ***n=*150** | ***n=*150** | ***n=*77** | ***n=*65** |
| Median [P25; P75] | 302.4 [0;4,149.6] | 0.0 [0.0; 1,778.4] | 4,149.6 [1,434.2; 14,820.0] | 2,470.0 [1,170.0; 6,916.0] |
| Mean (SD) | 6,110.7 (1,111.2) | 3,815.4 (1,731.8) | 11,824.7 (1,940.1) | 8,746.2 (3,901.7) |
| *Missing* | 1 | 1 | 0 | 0 |
| **Emergency consultations** | ***n=*150** | ***n=*150** | ***n=*39** | ***n=*37** |
| Median [P25; P75] | 0 [0;11.3] | 0 [0; 0] | 68.0 [31.8;327.9] | 87.4 [51.0;220.3] |
| Mean (SD) | 72.8 (265.3) | 37.2 (98.5) | 278.1 (464.3) | 149.6 (150.2) |
| *Missing* | 1 | 1 | 0 | 0 |
| **Supportive care** |  |  |  |  |
| **Nutritional support** | ***n=*150** | ***n=*150** | ***n=*127** | ***n=*70** |
| Median [P25; P75] | 96.0 [24.5;217.0] | 0 [0;48.0] | 132.3 [42.5;260.0] | 48.0 [25.8;115.2] |
| Mean (SD) | 161.9 (233.7) | 42.6 (85.2) | 189.9 (242.5) | 90.7 (105.5) |
| *Missing* | 1 | 1 | 0 | 0 |
| **Psychological treatment** | ***n=*150** | ***n=*150** | ***n=*15** | ***n=*14** |
| Median [P25; P75] | 0 [0;0] | 0 [0;0] | 26.7 [24.5;88.2] | 49.0 [35.0;88.2] |
| Mean (SD) | 7.2 (34.1) | 8.5 (46.5) | 71.3 (86.1) | 90.6 (129.0) |
| *Missing* | 1 | 1 | 0 | 0 |
| **Speech therapy** | ***n=*150** | ***n=*150** | ***n=*7** | ***n=*9** |
| Median [P25; P75] | 0 [0;0] | 0 [0;0] | 31.2 [7.8;35.1] | 23.4 [8.5;64.3] |
| Mean (SD) | 1.1 (6.2) | 2.5 (14.0) | 23.8 (17.5) | 40.7 (43.3) |
| *Missing* | 1 | 1 | 0 | 0 |
| **Physiatry, pain consultation, rehabilitation, tracheostomy, other** |  | ***n=*150** |  | ***n=*18** |
| Median [P25; P75] | N.A. | 0 [0;0] | N.A. | 89.0 [33.8;223.2] |
| Mean (SD) | N.A. | 2,389.2 (19,663.1) | N.A. | 20,940.8 (56,457.3) |
| *Missing* | N.A. | 1 | N.A. | 1 |

N.A.: Not applicable.

**Table S5.** Healthcare resource utilization among LA-HNSCC patients according to surgical status (n (%)).

| **HCR** | **Surgery (n=26)** | **No surgery (n=118)** | **Adjusted p value** |
| --- | --- | --- | --- |
| ST+RT | 20 (76.9) | 102 (86.4) | 0.6764 (Fisher's exact) |
| RT (no ST) | 4 (15.4) | 16 (13.6) | 1.0000 (Fisher's exact) |
| Outpatient Consultations | 26 (100) | 116 (99.1)^*^ | 1.0000 (Fisher's exact) |
| Imaging assessments | 26 (100) | 117 (100)^*^ | ---- |
| Laboratory | 26 (100) | 117 (100)^*^ | ---- |
| Biopsies, ECG, and others | 22 (84.6) | 108 (92.3)^*^ | 0.6764 (Fisher's exact) |
| Hospitalization | 19 (73.1) | 88 (75.2)^*^ | 1.0000 (Chi-squared) |
| Emergency consultations | 10 (38.5) | 41 (35.0)^*^ | 1.0000 (Chi-squared) |
| Nutritional support | 23 (88.5) | 102 (87.2)^*^ | 1.0000 (Fisher's exact) |
| Psychological treatment | 4 (15.4) | 20 (17.1)^*^ | 1.0000 (Fisher's exact) |
| Speech therapy | 7 (26.9) | 6 (5.1)^*^ | 0.0563 (Fisher's exact) |

^*^n=1 missing
